# Costs of colorectal cancer screening in Sweden: an observational, longitudinal cost description

**DOI:** 10.1136/bmjgast-2024-001574

**Published:** 2024-12-18

**Authors:** Naimi Johansson, Camilla Nystrand, Johannes Blom

**Affiliations:** 1 Department of Clinical Science and Education Södersjukhuset, Karolinska Institutet, Stockholm, Sweden; 2 Department of Learning, Informatics, Management & Ethics (LIME), Karolinska Institutet, Stockholm, Sweden; 3 Center for Health Economics, Informatics and Healthcare Research, Health Care Services Stockholm County (SLSO), Region Stockholm, Stockholm, Sweden; 4 Department of Surgery, Södersjukhuset, Stockholm, Sweden

**Keywords:** COLORECTAL CANCER, COLONOSCOPY, COLORECTAL CANCER SCREENING, COST-EFFECTIVENESS, SCREENING

## Abstract

**Objective:**

Colorectal cancer (CRC) screening programmes have been implemented worldwide, but the evidence of the economic consequences of screening programmes relies on data from short-term trials. The aim of this paper was to describe the costs of CRC screening in a population-based screening programme, using administrative real-world data. Specifically, we aimed to estimate the annual costs of the screening programme and the total costs of the full programme over five consecutive screening rounds.

**Methods:**

The CRC screening programme of Stockholm-Gotland, Sweden, targeted all resident men and women aged 60–69 years for biennial screening. The screening strategy was faecal occult blood testing (FOBT) sent to individuals’ home addresses, with a positive test result leading to an invitation to diagnostic colonoscopy. The cost description was conducted with a retrospective, bottom-up costing design from a healthcare perspective using (1) a prevalence-based approach and (2) an incidence-based approach, with two different study samples.

**Results:**

Annual healthcare costs were estimated using a sample of 124 608 individuals who were affected by the screening programme in 2017. Annual healthcare costs of the screening programme summed up to €273 758 per 10 000 people, equivalent to €27.4 per eligible individual. The sum of costs for colonoscopy procedures was more than two times as high as the costs for FOBT. The costs of the full screening programme were estimated using a cohort of 92 689 individuals who were invited to five consecutive rounds of screening between 2009 and 2021. Total healthcare costs over five screening rounds were €960 654 per 10 000 people, equivalent to €96.1 per individual.

**Conclusion:**

The costs of diagnostic colonoscopies for a minority of participants were driving the costs of the CRC screening programme. The ongoing population-based screening programme and high-quality individual level data with long-term follow-up provide the opportunity to thoroughly describe the costs of CRC screening.

**WHAT IS ALREADY KNOWN ON THIS TOPIC**

- Previous evidence from health economic models make extrapolations about future costs based on short-term trials, but there is lack of economic evidence from long-term individual-level follow-up of colorectal cancer screening.

**WHAT THIS STUDY ADDS**

- Using administrative data from a population-based colorectal cancer screening programme, this study provides a thorough cost description of the various cost components, development over time and distribution of costs by results category.

**HOW THIS STUDY MIGHT AFFECT RESEARCH, PRACTICE OR POLICY**

- Evidence on economic consequences alongside the health benefits of an intervention is fundamental for addressing ethically complex questions in health policy. Real-world longitudinal individual-level data used in the present study confirm the results from previous research.

## Introduction

 With an incidence of 1.9 million new cases of colorectal cancer (CRC) in 2020, CRC is the third most common cancer worldwide and in 2040, the CRC burden is estimated to 3.2 million new cases and 1.6 million disease-specific deaths.^1^ CRC is among the most resource consuming cancers, followed by lung, breast and prostate cancer.^2^ The economic burden of CRC across Europe has been estimated to €19.1 billion in 2015.^3^ Secondary prevention of CRC with population-based screening has the potential to reduce deaths from the disease by detecting the cancer at an early curable stage. As both larger potential precursors like adenomatous polyps and cancer may bleed, faecal occult blood tests (FOBT) are commonly used as primary screening tests, followed by a diagnostic colonoscopy when positive. In 2003, the European Commission issued recommendations to screen for CRC with FOBT in men and women aged 50–74 years,^4^ and population-based screening was implemented 2008 in the region of Stockholm-Gotland, Sweden.^5^ The Stockholm-Gotland screening programme has shown to decrease CRC mortality by 14%.^6^

Up to 80 cost-effectiveness analyses of CRC screening from various settings have been documented in recent reviews, often concluding that CRC screening is cost-effective compared with no screening.^7 8^ Previous cost-effectiveness analyses included the costs of screening along with the costs of CRC treatment, however, only few have in detail described the costs of FOBT and diagnostic colonoscopies.^9^,^11^ Other studies have specifically conducted budget impact analyses and presented costs of CRC screening programmes, usually in a healthcare payer perspective.^12^,^14^ A methodological review of budget impact analyses of cancer screening identified six studies on CRC screening.^15^ The common methodological approach across previous cost-effectiveness and budget impact analyses is health economic modelling, making extrapolations about future resource use based on data from short-term RCTs and best available evidence from literature.^7 15^ Exceptions include recent studies on costs of screening for subpopulations in Iran (19 000 individuals) and in the US (333 000 individuals).^16 17^

Taken together, there is a lack of economic evidence from long-term individual-level follow-up of CRC screening. The aim of this paper was to describe the costs of CRC screening in a population-based programme, using administrative real-world data. Specifically, we aimed to estimate the annual costs of the screening programme, and the total costs of the full programme inviting individuals over five consecutive screening rounds.

## Methods

The study was approved by the Ethics Review Board of Sweden (#2020–06757) and in accordance with the Declaration of Helsinki. Informed consent was waived because data were pseudonymised. The reporting follows the Consolidated Health Economic Evaluation Reporting Standards (CHEERS) checklist (online supplemental file 2).

### The screening program

The CRC screening programme of Stockholm-Gotland, covering approximately 25% of the Swedish population, aimed to invite all resident men and women aged 60–69 years for screening every second year.^5^ No exclusions. In the start of the programme, two or three birth cohorts in the target group were invited each year (by randomisation), consecutively rolling out the programme to eligible birth cohorts. Each individual was invited up to five screening rounds. The programme was centrally administered by the Regional Cancer Center (RCC) Stockholm-Gotland, sending letter invitations including screening test kits and instructions to residents’ home addresses with a prepaid return envelope. Initially, guaiac FOBT (gFOBT) was used, but in September 2015, the gFOBT was changed to a faecal immunochemical test (FIT) with gender-specific cut-off levels of blood in the stool for a positive test.^18^ A positive test result; at least one out of the two panels of the three test cards positive with gFOBT, or 40 µg Hb/g faeces (women) and 80 µg/g (men) or more with FIT; led to an invitation to colonoscopy at an endoscopy unit (participants allocated by catchment area). In 2019, national implementation of the CRC screening programme started, by recommendation of the Swedish National Board of Health and Welfare targeting the broader age group 60–74-year olds.^19^

### Analytical approach

This analysis of the costs of CRC screening builds on the analytical perspectives of a cost-of-illness analysis—an observational cost description of the economic burden to society of the screening programme.^20^ The analysis was restricted to activities and resource use directly related to the screening process, excluding costs related to CRC treatment or complications. The analysis was conducted with a retrospective, bottom-up costing design primarily from a healthcare perspective using (1) a prevalence-based approach and (2) an incidence-based approach.^21^ A prevalence based approach aims to estimate costs of all cases in a given time period, while an incidence-based approach aims to estimate lifetime costs of new cases arising in a time period.^21^ The healthcare perspective was chosen considering the healthcare sector being the one primarily affected by the screening process and considering access to reliable individual-level data of healthcare resource use. We also considered a wider societal perspective including costs borne by participants, municipalities and society at large. However, due to lack of individual data, the societal perspective was seen as more explorative.

In the prevalence-based approach, we estimated annual costs of screening taking into account of resource use for all screening activities occurring in a given year, defined by when in time a test kit was sent out/analysed or a colonoscopy was conducted (irrespective of individual’s year of invitation or screening round).^21^ To estimate the annual costs of screening in one year, no discounting was needed. In the incidence-based approach, we estimated costs of the full screening programme over five rounds, taking account of resource use of individuals invited up to five consecutive rounds.^2^ The costs of the full screening programme over five rounds were discounted to present value (year 2023) taking into account of costs occurring up to 9 years in the future (the fifth screening round), with a discount rate of 3% as recommended by national guidelines.^21 22^ All costs were converted to Euros (€1=11.48 Swedish krona), price level of 2023.

### Data and study samples

Register data were retrospectively retrieved from the regional Screening Register of RCC Stockholm-Gotland (years 2008–2019) and the national Swedish Register for Colonoscopy and Colorectal Cancer Screening of the RCC of Sweden (SveReKKS, years 2019–2021).^23 24^ The two registers include information about each individual invited to screening, their stool tests, FOBT analysis results and colonoscopy outcomes.

With the aim to estimate (1) the annual costs of the screening programme and (2) the costs of the full programme over five rounds, two separate study samples were used. The sample used for estimating the annual costs was a cross-section defined from a dataset of all individuals invited for screening with FIT in 2015–2017, which was the first years of FIT replacing gFOBT. We restricted this dataset to all screening activities in 2017. Hence, the final sample for analysis included individuals born 1948–1957 resident in Stockholm-Gotland, who received an invitation to screening in 2016 or 2017 with screening activities in 2017.

The second sample used for estimating the costs for the full programme over 5 years was a longitudinal cohort defined from a dataset of individuals born 1938–1954, invited in the roll out of the screening programme starting 2008. We restricted this dataset to individuals born 1949–1952, who were the first four birth cohorts to be invited to five consecutive rounds of screening, from 2009 to 2021. Each birth cohort was invited for a first round at age 60 (first round at 62 years for individuals born in 1951), and thereafter invited every second year. The birth cohorts in the full dataset invited to four or less screening rounds (born in 1938–1948, and 1953–1954) were excluded from analyses.

### Identifying, measuring and valuing costs

The registers enabled bottom-up identification and measurement of each individual screening occasion; the number and dates of FOBT, retests, lab analyses, reminders, test results letters, diagnostic colonoscopies with and without biopsy or polypectomy, pathological-anatomical diagnosis, remittance to CT, recommendations of adenoma surveillance programme as well as colonoscopy outcomes. Other healthcare resources identified outside the scope of the registers were resources used for test administration and nurse contact phone calls preceding colonoscopy and bowel preparation. Following Swedish recommendations, each colonoscopy conducted was assumed to be preceded by a 20 min phone call with the endoscopy unit and one dose bowel preparation (macrogol, *eg,* Plenvu).^25^

Several sources were used to retrieve a relevant valuation (unit cost) of each resource, presented in online supplemental appendix table A1. Unit costs of the costs of FOBT, including invitation letters, reminders and lab analyses were retrieved from RCC Stockholm-Gotland administration (personal conversation). The mean cost of administration was estimated by the total costs of RCC’s test administration of the national programme, divided by the number of individuals in the population. Costs of hospital procedures were collected from the Swedish Cost per patient (KPP) database.^26^ Costs of bowel preparation drugs were collected from the reimbursement and price database of the Swedish Dental and Pharmaceutical Benefits Agency.^27^ For valuation of contact phone calls, the national average wage for nurses including fees for social benefits was used, collected from Statistics Sweden.^28^

From a broader societal perspective, relevant costs to include are those borne by participants, municipalities and society at large. Identified costs were related to the colonoscopy procedure. Relevant resources and private costs for colonoscopy participants were costs for transportation and time (loss of spare time); for municipalities costs for mobility service assistance for colonoscopy participants living in long-term care facilities; and for society at large through loss of productivity due to work absence among colonoscopy participants in the workforce.

Patients’ costs for transportation were estimated by two times (roundtrip) the mean distance to an emergency hospital in Sweden, 14.2 km,^29^ multiplied by €0.22 (per kilometre tax-deductible set by the Swedish Tax Agency). We assumed 1.2% of colonoscopy participants were in need of mobility service assistance (proportionate to the population share 65–79-year olds, residing in long-term care^30^). Measuring hours of mobility service assistance, we used a patient time diary estimate of 4.45 hours of dedicated time including roundtrip travelling, bowel preparation and colonoscopy procedure, estimated by Jonas *et al*.^31^ For valuation of mobility service assistance, the national average wage for care assistants was used.^28^ Patients’ willingness to pay to avoid the loss of time and the discomfort associated with colonoscopy has been estimated to US$263, equivalent to €241 in prices of 2023.^32^ The willingness-to-pay estimate includes the value of the income loss for patients in the workforce, hence accounting for the loss of productivity to society.

To assess the robustness of the results, a deterministic sensitivity analysis was conducted varying one parameter at a time.

### Statistics

Descriptive statistics presented as percentage, mean, SD and sum as applicable.

## Results

### The annual costs of screening

The annual costs of screening were estimated taking account of resource use for all screening activities occurring in 2017. The left panel of table 1 presents descriptive statistics of the 124 608 individuals who were affected by the screening programme in 2017. Among the affected individuals, 8.5% were invited in 2016 but had reminders sent, tests analysed or colonoscopies performed in 2017. The testing yielded 64.5% negative results, 1.1% positive test but without findings in colonoscopy (*ie,* false positives; defined by register indicator *Return to screening programme*), 0.7% were found to have CRC or an advanced adenoma (true positives), and 0.2% had a positive test but non-compliant with colonoscopy. Hence, a total of 67.3% of affected individuals participated in testing, while the remaining 32.7% and 0.7% were non-participants or had an incomplete test, respectively.

**Table 1:** Descriptive statistics of the two samples

2017 sample			1949–1952 cohort					
124 608 individuals and screening occasions			92 689 individuals			427 695 screening occasions		
	n	%		n	%		n	%
Sex			Sex					
Men	61 469	49.33	Men	45 596	49.19			
Women	63 139	50.67	Women	47 093	50.81			
Birthyear			Birthyear			Year		
1947	3	<0.00	1949	24 050	25.95	2009	23 216	5.43
1948	1866	1.50	1950	23 623	25.49	2010	23 310	5.45
1949	21 470	17.23	1951	22 112	23.86	2011	23 226	5.43
1950	1932	1.55	1952	22 904	24.71	2012	45 707	10.69
1951	20 938	16.80				2013	44 303	10.36
1952	2255	1.81	Censored			2014	44 069	10.30
1953	22 303	17.90	Deceased (2009–2021)	8326	8.98	2015	42 549	9.95
1954	2406	1.93	Emigrated (2009–2021)	2269	2.45	2016	42 277	9.88
1955	23 466	18.83				2017	40 816	9.54
1956	2703	2.17	Year of first invitation			2018	40 556	9.48
1957	25 266	20.28	2009 (b 1949)	24 050	25.95	2019	19 373	4.53
Year of invitation			2010 (b 1950)	23 623	25.49	2020	19 706	4.61
2016	10 673	8.57	2012 (b 1952)	22 904	24.71	2021	18 587	4.35
2017	113 935	91.43	2013 (b 1951)	22 112	23.86			
Invited for screening round			Number of screening occasions invited			Screening round		
First	27 969	22.45	1	3200	3.45	Round 1	92 693	21.67
Second	48 178	38.66	2	3483	3.76	Round 2	89 491	20.92
Third	25 059	20.11	3	3859	4.16	Round 3	86 006	20.11
Fourth	1932	1.55	4	4785	5.16	Round 4	82 146	19.21
Fifth	21 470	17.23	5	77 360	83.46	Round 5	77 359	18.09
Participation and test results*			Participation and test results of each individual			Participation and results of each screening occasion		
CRC or advanced adenoma†	873	0.70	CRC or advanced adenoma†	1642	1.77	CRC or advanced adenoma†	1642	0.38
False positive†	1397	1.12	False positive†	3842	4.15	False positive†	4107	0.96
Positive, no colonoscopy	290	0.23	Positive, no colonoscopy	690	0.74	Positive, no colonoscopy	800	0.19
Negative test	80 393	64.52	Negative test	64 524	69.61	Negative test	258 682	60.48
Incomplete test	909	0.73	Incomplete test	645	0.70	Incomplete test	3025	0.71
Non-participant	40 746	32.70	Non-participant	21 346	23.03	Non-participation	159 439	37.28

* Categorizsation of individuals taking account of activities 2016–2018.

† Defined by register indicator for Return to screening programprogramme.

CRCcolorectal cancer

Table 2 presents resource use and annual costs of screening in means (SD) and per 10 000 people (online supplemental appendix table A2 presents the distribution of costs). Resources for testing included 9259 test kits, 6570 lab analyses and 6084 result letters per 10 000 people, while resources for colonoscopy concerned a much smaller group—only 161.9 (60.4+8.1+93.3) colonoscopy procedures per 10 000 people. Colonoscopy with polypectomy was the most common type of procedure (93.3 per 10 000 people). In monetary terms, the sum of costs for colonoscopy procedures were more than two times as high as the sum of costs for FOBT, although it only applied to 1.8% of the invited population. Lab analyses and test kits made up the bulk of costs for FOBT, a total of €84 646 per 10 000 people. Costs for ‘simple’ colonoscopies without findings and colonoscopies with polypectomy contributed to the majority of costs for colonoscopy procedures, which amounted to €189 112 per 10 000 people. Hence, annual healthcare costs of the screening programme summed up to €273 758 per 10 000 people, equivalent to €27.4 per eligible individual, where 69% were costs for colonoscopies.

**Table 2:** Average annual resource use and costs of screening

	Resource use			Annual costs (€), non-discounted		
	Mean (item/ person)	SD	Item/10 000 people	Mean (€/person)	SD	€/10 000 people
Fixed costs of admin RCC	–	–	–	0.94	0.00	9445.37
Test kits, gFOBT*	–	–	–	–	–	–
Test kits, FIT*	0.926	0.300	9259.52	2.58	0.84	25 786.06
Re-test kits	0.028	0.196	280.08	0.08	0.55	779.97
Reminders	0.379	0.493	3791.33	0.30	0.38	2956.68
Lab analyses	0.657	0.524	6570.12	4.09	3.26	40 932.61
Test results letters	0.608	0.488	6084.52	0.47	0.38	4745.03
**Sum of costs for FOBT**	–	–	–	**8.46**	**3.81**	**84 645.71**
Phone contacts	0.016	0.126	161.87	0.18	1.38	1763.57
Bowel preparation	0.016	0.126	161.87	0.18	1.41	1808.59
Colonoscopy without findings	0.006	0.078	60.43	4.57	58.72	45 720.26
Colonoscopy with biopsy	0.001	0.028	8.11	0.74	25.98	7399.50
Colonoscopy with polypectomy	0.009	0.096	93.33	9.70	99.92	96 988.08
Findings sent to PAD†	0.007	0.085	73.59	–	–	–
Remittance to CT scans	0.0002	0.013	1.77	0.10	7.16	950.91
Adenoma surveillance programme‡	0.005	0.071	50.64	3.45	48.33	34 481.45
**Sum of costs for colonoscopy**	–	–	–	**18.91**	**156.43**	**189 112.40**
**Sum of healthcare costs**	–	–	–	**27.38**	**156.55**	**273 758.10**
*Related to colonoscopy visits:*						
Patient transportation cost§	0.016	0.126	161.87	0.10	0.78	1001.40
Patient value of time and disc.§	0.016	0.126	161.87	3.90	30.50	39 045.06
Mobility service assist (hours)¶	0.0009	0.007	8.64	0.02	0.14	184.10
**Sum of costs to wider society**	–	–	–	**4.02**	**31.43**	**40 230.56**
**Total costs societal perspective**	–	–	–	**31.40**	**186.49**	**313 988.60**

The lines marked by bold represent the sum of the values above.

* gFOBT was used up until 2015, after which FIT was used.

† Costs for pathological-anatomical diagnosis (PAD) were included in the average cost for colonoscopy with biopsy and polypectomy.

‡ Assuming 90% compliance adenoma surveillance colonoscopy,^40^ three3 years after initial diagnostic colonoscopy.

§ Each colonoscopy was assumed to involve patient travelling and patient’s opportunity cost of loss of time and discomfort.

¶ Mobility service assistance for colonoscopy participants residing in long-term care facilities.

FITfaecal immunochemical testgFOBTguaiac faecal occult blood testingRCCRegional Cancer Center

Considering a broader societal perspective added another €40 231 per 10 000 people, where participants’ own valuation of time and discomfort of colonoscopy was the main contributor. Summing up, in a societal perspective, the annual costs of the screening programme were €313 989 per 10 000 people, or €31.4 per individual. In the region of Stockholm-Gotland, the annual healthcare costs of CRC screening corresponded to about €3.4 million for the 124 608 affected individuals in our sample, and in annual societal costs about €3.9 million.

**Figure 1:**
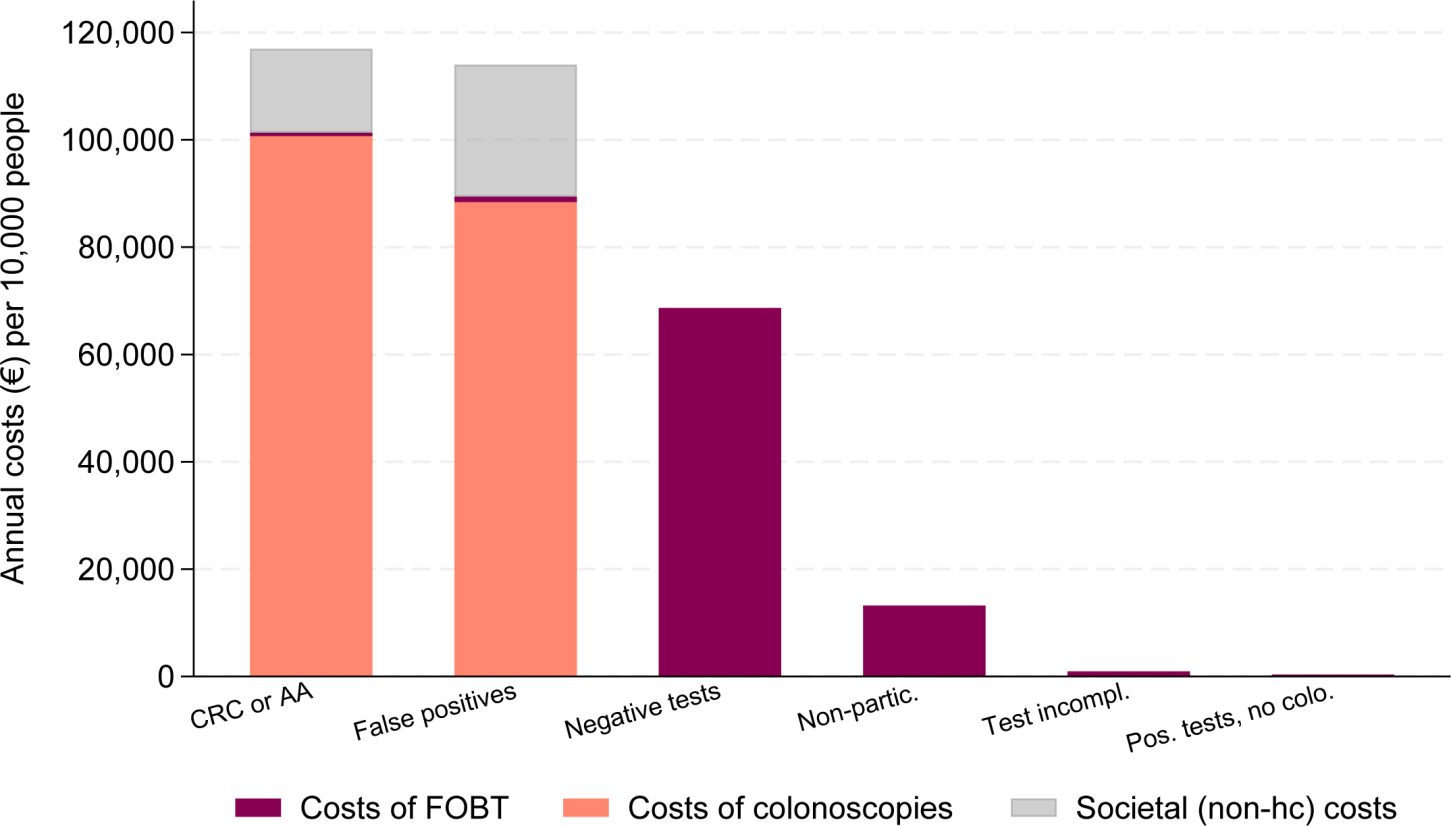
Annual costs per 10 000 people, by participation and results category. The number of people per 10 000 in each category were CRC or AA 70 individuals; false positives 112 individuals; negative tests 6452 individuals; non-participants 3270 individuals; test incomplete 73 individuals; and positive test no colonoscopy 23 individuals. CRC or AA (true positive) defined as finding of colorectal cancer or advanced adenoma at colonoscopy; false positive defined as a positive test but without findings at colonoscopy; negative test defined as negative result from FIT; non-participant defined as no test returned for analysis; test incomplete defined as test returned but infeasible for analysis; positive test but no colonoscopy defined as a positive result from FIT but non-compliant to colonoscopy. CRC, colorectal cancer; FIT, faecal immunochemical test; FOBT, faecal occult blood testing.

Disaggregating the annual healthcare costs of screening to groups by participation and test status, the major part of costs concerned colonoscopy procedures for the relatively small groups of true positives (70 of 10 000) and false positives (112 of 10 000), €101 328 and €89 407 per 10 000 people, respectively (figure 1 and online supplemental appendix table A3). The large group of individuals with negative tests (6452 of 10 000) together contributed €68 645 per 10 000 people in costs for FOBT only.

### The costs of the full screening programme over five rounds

The costs of the full screening programme were estimated accounting for all resource use for a sample of individuals invited up to five consecutive screening rounds. The right panel of table 1 presents descriptive statistics of 92 689 individuals and 427 695 screening occasions for the birth cohorts 1949–1952. In the sample, 77 360 individuals (83.5%) were invited to five consecutive screening rounds. Confirmed CRC or advanced adenoma (referred to an adenoma surveillance programme), emigration, moving to another region in Sweden, or death were reasons for not being reinvited to screening. Costs of deceased (9.0%) and emigrated (2.5%) individuals were included up until the event. Considering invitations in up to five screening rounds, 76.3% (1.8+4.2+0.7+69.6) of individuals participated in at least one screening round. Overall, 4.2% of individuals had a false-positive test, 1.8% were found to have CRC or an advanced adenoma, and 0.7% had a positive test but were non-compliant with colonoscopy. Considering all 427 695 screening occasions of the sample, 60.5% resulted in a negative test and 37.3% were non-participation occasions.

Individuals used on average 4.6 test kits (2.6 gFOBTs and 2.0 FITs) over the full screening programme (table 3). The patterns of resource use and costs were similar in the costs of the full screening programme compared with annual costs of screening, with colonoscopy procedures concerning a small group (625 colonoscopies per 10 000 people) but contributing to a large part of the costs. For the full screening programme, costs for FOBT were in present value €370 852 per 10 000 people and costs for colonoscopy procedures were €589 802. Hence, total healthcare costs of the full screening programme were €960 654 per 10 000 people, where 61% were costs for colonoscopies. Including the broader societal perspective added €137 151 per 10 000 people, to a total of €1 097 805 per 10 000 people for the full screening programme over five rounds, equivalent to €109.8 per individual (online supplemental appendix table A2 presents the distribution of costs). For an average size birth cohort in the region of Stockholm-Gotland, about 23 000 individuals, the healthcare costs of the full screening programme over five rounds would correspond to €2.2 million in present value, and the societal costs of the full screening programme to €2.5 million in present value.

**Table 3:** Average resource use and costs for five-round screening programme

	Resource use			Discounted costs (€)		
	Mean (item/person)	SD	Item/ 10 000 people	Mean (€/person)	SD	€/10 000 people
Fixed costs of admin RCC	–	–	–	3.68	0.74	36 759.42
Test kits, gFOBT*	2.628	0.872	26 282.95	9.14	2.81	91 401.76
Test kits, FIT*	1.986	1.040	19 860.07	4.62	2.48	46 218.93
Re-test kits	0.213	0.618	2131.65	0.67	1.97	6716.64
Reminders	2.187	1.876	21 873.25	1.46	1.23	14 637.57
Lab analyses	2.894	2.032	28 937.85	17.36	12.25	173 559.60
Test results letters	2.862	2.040	28 615.15	1.83	1.33	18 276.79
**Sum of costs for FOBT**	–	–	–	**37.09**	**13.88**	**370 851.50**
Phone contacts	0.062	0.257	624.56	0.78	3.21	7787.54
Bowel preparation	0.062	0.257	624.56	0.67	2.74	6655.64
Colonoscopy without findings	0.029	0.175	285.69	18.96	116.55	189 554.10
Colonoscopy with biopsy	0.004	0.063	39.06	3.17	50.92	31 689.02
Colonoscopy with polypectomy	0.030	0.172	299.82	24.48	141.10	244 784.70
Findings sent to PAD†	0.029	0.192	288.71	–	–	–
Remittance to CT scans	0.001	0.031	9.28	0.74	20.99	7372.45
Adenoma surveillance programme‡	0.014	0.118	141.44	7.40	61.94	73 970.78
**Sum of costs for colonoscopy**	–	–	–	**58.98**	**250.82**	**589 802.10**
**Sum of healthcare costs**	–	–	–	**96.07**	**251.83**	**960 653.60**
*Related to colonoscopy visits:*						
Patient transportation cost§	0.062	0.257	624.56	0.34	1.41	3413.91
Patient value of time and disc§	0.062	0.257	624.56	13.31	54.87	133 109.70
Mobility service assist (hours)¶	0.003	0.014	33.35	0.06	0.26	627.64
**Sum of costs to wider society**	–	–	–	**13.72**	**56.53**	**137 151.20**
**Total costs societal perspective**	–	–	–	**109.78**	**306.08**	**1,097,805.00**

* gFOBT was used up until 2015, after which FIT was used.

† Costs for pathological-anatomical diagnosis (PAD) were included in the average cost for colonoscopy with biopsy and polypectomy.

‡ Assuming 90% compliance adenoma surveillance colonoscopy,^40^ three3 years after initial diagnostic colonoscopy.

§ Each colonoscopy was assumed to involve patient travelling and patient’s opportunity cost of loss of time and discomfort.

¶ Mobility service assistance for colonoscopy participants residing in long-term care facilities.

FITfaecal immunochemical testgFOBTguaiac faecal occult blood testingRCCRegional Cancer Center

**Figure 2:**
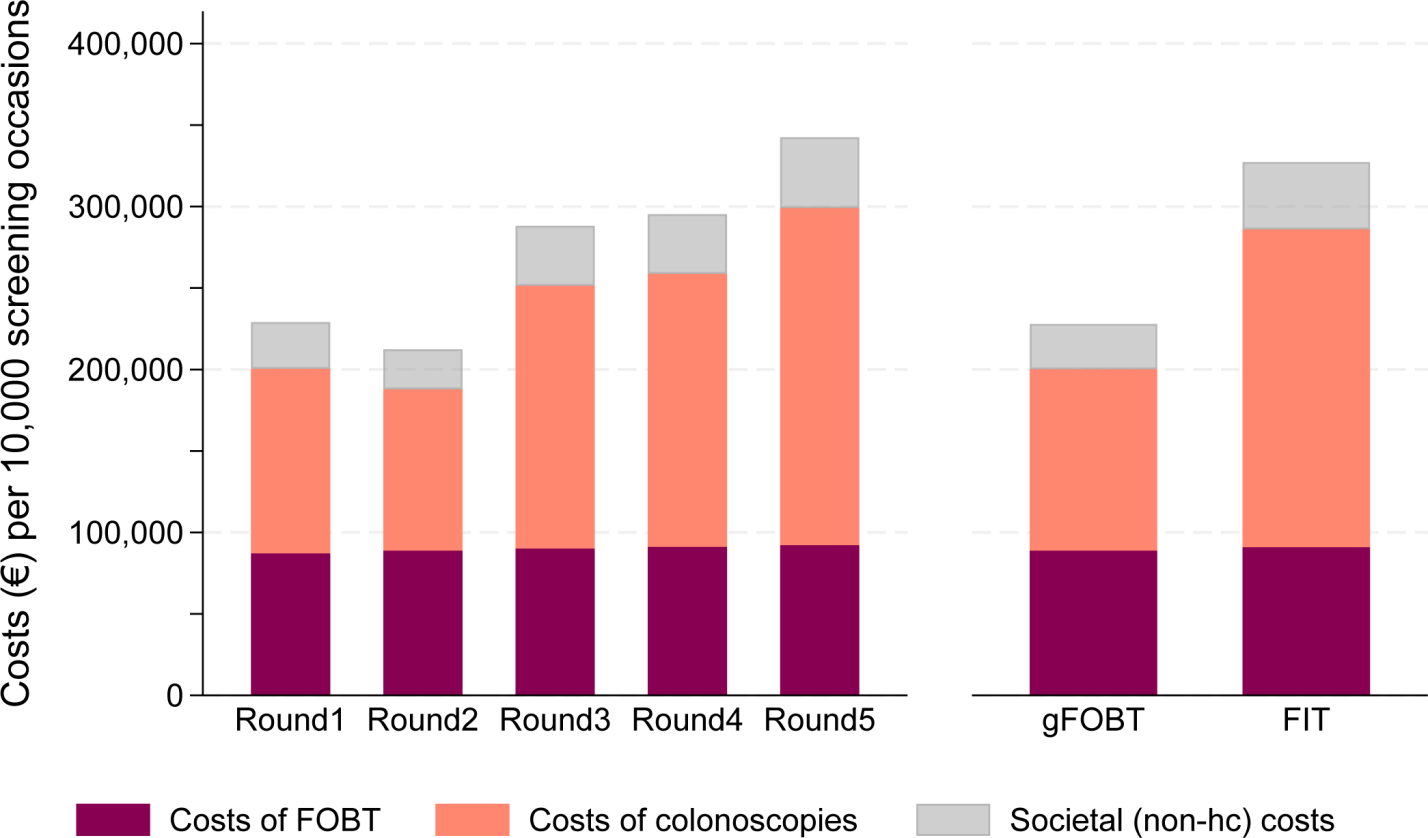
Costs (€) per 10 000 screening occasions (invited individuals), comparison by screening round and by type of FOBT. Costs are not discounted in this comparison. FIT, faecal immunochemical test; gFOBT, guaiac faecal occult blood testing.

Notably, there was a change in resource use and increased costs over time, screening round and type of FOBT used. Screening rounds 3, 4 and 5 had fewer reminders and retest kits sent out, but more lab analyses and colonoscopies were conducted compared with earlier screening rounds (online supplemental appendix table A4). The increase in colonoscopies was primarily seen in colonoscopies with polypectomy, while colonoscopies without findings or with biopsy remained stable, resulting in higher mean cost per screening occasion (figure 2). The pattern was repeated when subcategorising by type of FOBT. Specifically, there were 40 colonoscopies with polypectomy per 10 000 people among screening occasions with gFOBT, but 97 colonoscopies with polypectomy per 10 000 people screened with FIT (online supplemental appendix table A4). The healthcare costs were €200 129 per 10 000 screening occasions (invited individuals) with gFOBT, and €285 955 per 10 000 screening occasions with FIT (figure 2).

### Sensitivity analysis

The deterministic sensitivity analysis showed that the valuation of colonoscopy (with and without biopsy or polypectomy) had the highest impact on the results. A 20% higher valuation of colonoscopy would result in annual healthcare costs of €31.1 per person and for the full screening programme healthcare costs of €107.5 per person (online supplemental appendix figures A1 and A2). Varying the valuations of lab analyses and FOBT, and the assumptions on compliance to and timing of adenoma surveillance colonoscopy had a smaller impact on the healthcare costs of screening. Finally, including complications of colonoscopy as indicated in the registers among about 0.008 of colonoscopy participants with a valuation of €6650 per complication (€5157 in Arrospide *et al*,^9^ adjusted to 2023 price level) had a minor impact on the results. Increasing the rate and the valuation of complications by 20% yielded €27.5 per person in annual healthcare costs and €96.3 per person for the full five-round screening programme.

## Discussion

### Costs of colonoscopy

In this paper, we have conducted an observational cost description of CRC screening in a population-based screening programme in Sweden, using administrative real-world data. The study presents economic evidence of the CRC screening programme in two aspects: annual costs of screening and full costs of five consecutive screening rounds. The estimated annual healthcare costs of the screening programme were €273 758 per 10 000 people, equivalent to €27.4 per eligible individual. Considering the full programme over five screening rounds, the estimated healthcare costs were €960 654 per 10 000 people, or €96.1 per individual. The costs of screening were driven by the high costs of colonoscopy procedures, despite quite a small group being affected.

The results of the full five-round screening programme are comparable to a previous budget impact model of the English eight-round screening programme where costs of screening were estimated to €125–€168 per individual depending on FOBT type (results converted to Euros 2023 for comparability).^10^ The higher costs can partly be explained by the length of the programme, and by a higher rate of adenoma surveillance colonoscopies in the English model. Direct comparison to other previous studies is difficult due to lack of detail or results presented only as the total costs over a running long-term period.^9 11^ The findings of the current cost description can serve as reliable input in future cost-effectiveness and budget impact models of various CRC screening strategies.

### Ethical considerations

The inherent ethical dilemma of any screening programme is the fact that a large group of the population is invited to participate, while a minority of individuals will benefit from the screening. On the individual level, there is an ethical dimension to why individuals should participate in an intervention they may have no benefit from. On the societal level, it is ethically questionable whether society ought to allocate (public) economic resources to an intervention targeted at an overall healthy population. To support decision-making, the additional costs need to be directly related to the health benefit of the intervention, in other words, assessed in a cost-effectiveness analysis.

The results from the current study showed that out of €273 758 annual healthcare costs per 10 000 persons, €101 328 (37%) were attributed to the group of true positives (CRC or advanced adenoma). The remaining costs fell in the groups of false positives (33%), negative tests (25%) and non-participants (5%)—costs that potentially could be reduced with a risk-stratified screening programme to avoid testing true negatives, or with fine-tuning testing techniques to avoid false positives. The FIT cut-off levels of blood in the stool provide such an opportunity to adjust the positivity rate, where a lower cut-off (eg*,* 40 µg Hb/g faeces) yield a higher positivity rate but decrease the specificity of the test (more false positives). On the other hand, a higher cut-off leads to fewer CRC cases detected.^33^ Whether the benefits of earlier disease detection and potential cost-savings of screening outweigh the increased costs related to unnecessary colonoscopies of false positives, and the increased worry related to a false-positive result,^34 35^ needs further investigation. The detailed subcategorisation of the costs of screening in the current study implies that further developments of screening strategies such as risk-stratification or improved test specificity has the potential to markedly reduce costs, freeing up economic resources for other healthcare interventions.

### Additional findings

An interesting finding of the costs of the full five-rounds-screening-programme was the increased costs over screening round, with subsequently growing colonoscopy costs for screening round 3, 4 and 5, due to higher rates of polypectomies. This finding has several potential explanations, as the order of screening round inevitably is associated with participants age, calendar time, and in our study period, the change from gFOBT to FIT. Older age has been shown associated with increased polyp findings.^36^ The change to FIT in 2015 resulted in increased uptake and the positivity rate among participants increased from 1.81% with gFOBT to 2.57% with FIT, which will have a direct effect on costs of the additional diagnostic colonoscopies.^18^ Other changes over calendar time could be improvements in endoscope equipment, or improved adherence to recommendations to remove all larger polyps,^25^ leading to higher rates of polyp findings and polypectomies, and thereby costs.

Considering the broader societal perspective, the results from our study showed that the healthcare system carried the largest part of costs for CRC screening, while the costs to the wider society made up about 12% of total societal costs. This contrasts the economic burden of CRC *treatment* where the major part of costs falls outside the healthcare system as production loss and costs of informal care, estimated to 61% on average in Europe and 78% in Sweden.^3^ In our study, the major driver of the costs to the wider society (non-healthcare costs) was the willingness-to-pay estimate of patients’ value of time and discomfort of colonoscopy, which accounts for the value of production loss to society. An alternative would have been to apply the commonly used human capital approach to estimate society’s production loss; however, the human capital approach may overestimate the value of time when it comes to procedures involving irregular preparation (and recovery) such as colonoscopy.^32^

### Limitations

A limitation to the study was that measurements of nurse contact calls and of the use of bowel preparation drugs preceding colonoscopy were not available in the screening register. However, these costs are minor in comparison to other identified resources. Similarly, there was limited information about individual recommendations and adherence to adenoma surveillance colonoscopies. The deterministic sensitivity analysis showed that despite simplifying assumptions, the costs of adenoma surveillance had a small impact on the healthcare costs of screening.

Another limitation was that the screening registers did not provide (full) information on other potential consequences of screening, such as complications of colonoscopy, bowel preparation and postprocedure visits due to anxiety, which implies that the healthcare costs were underestimated. In a review on psychological effects of CRC screening, three of the seven studies showed evidence of higher psychological distress among participants with false-positive results compared with participants with negative test results shortly before and/or after colonoscopy.^37^ However, to the best of our knowledge, only a few previous studies have investigated screening consequences related to healthcare use and costs.^38^ Results from a Danish lung cancer screening trial showed significantly increased number of outpatient visits, non-surgical procedures and higher costs for psychologist visits for screening participants compared with a non-screened randomised control group.^39^ There is a need to further investigate to what extent anxiety and psychological distress following CRC screening may impact use and costs of healthcare.

The screening registers provided some information about complications of colonoscopy, which enabled inclusion of complications in the sensitivity analysis. The sensitivity analysis indicated that costs of complications were minor, which was similar to previous results from the UK that showed that costs of perforation and admissions due to bleeding contributed to less than 1% of screening costs.^10^ To fully capture the consequences of screening on healthcare use and costs, there is need to collect more detailed data, which can be achieved by linking the Swedish screening registers to registers of inpatient admissions and outpatient care contacts.

## Conclusion

To the best of our knowledge, the current study is the first to provide economic evidence with long-term individual-level follow-up of CRC screening. The use of administrative real-world data covering up to 13 years of follow-up and collected in the context of a Swedish CRC screening programme implemented in clinical practice strengthens the validity and reliability of the results. The findings’ generalisability applies to other healthcare settings where population-based CRC screening is offered through FOBT followed by diagnostic colonoscopy. Under the assumption of similar resource use, researchers may apply their country-specific valuations (unit costs) to estimate expected costs of screening and cost-effectiveness of various screening strategies in their setting.

In conclusion, the current study showed that the costs of diagnostic colonoscopies for a minority of participants were driving the costs of the CRC screening programme. The ongoing population-based screening programme together with high-quality real-world data provides the opportunity to describe the costs of CRC screening with a high level of detail.

## supplementary material

10.1136/bmjgast-2024-001574 online supplemental file 1

10.1136/bmjgast-2024-001574 online supplemental file 2

## Data Availability

Data may be obtained from a third party and are not publicly available.
