# Supplementary material for: Costs of colorectal cancer screening in Sweden: an observational, longitudinal cost description
Source: BMJ Open Gastroenterol. 2024 Dec 18;11(1):e001574. doi: 10.1136/bmjgast-2024-001574 (PMC11667418; doi:10.1136/bmjgast-2024-001574)
Supplement: online supplemental file 1 [file bmjgast-11-1-s001.pdf]

Costs of colorectal cancer screening in Sweden
# – an observational longitudinal cost description

APPENDIX

Table of Contents

Table A1. Unit costs 1
Table A2. Distribution of costs 2
Table A3. Annual costs of screening, grouped by participation-test results category 3
Table A4. Five-rounds-screening program: Selected resources 4

Figure A1. Deterministic sensitivity analysis – Annual costs of screening. 5
Figure A2. Deterministic sensitivity analysis – Costs for five-rounds-screening program. 6

Table A1. Unit costs

	SEK 2023	EUR 2023	Source
Fixed administrative costs of testing	10.84	0.94	RCC
Test kit gFOBT	39.07	3.40	RCC
Test kit FIT	31.96	2.78	RCC
Letter (reminder/test result)	8.95	0.78	RCC
Lab analysis of test	71.50	6.23	RCC
Hourly wage, nurse (contact call)	375.86	32.75	SCB [28]
Bowel preparation drugs [a]	128.23	11.17	TLV [27]
Colonoscopy (without findings)	8,683.00	756.59	KPP [26]
Colonoscopy with biopsy	10,477.00	912.91	KPP [26]
Colonoscopy with polypectomy	11,926.00	1,039.17	KPP [26]
CT-colon	6,868.00	598.44	KPP [26]
Transportation cost (roundtrip)	71.00	6.19	Avdic [29]
Patient's WTP to avoid colonoscopy [b]	2,768.32	241.22	Jonas et al. [32]
Hourly wage, care assistant	244.44	21.30	SCB [28]

[a] One dose of Plenvu®.

[b] Willingness to pay (WTP) estimate of US$263 in 2006, converted to Swedish krona and adjusted for Swedish inflation (Turner et al. 2019 *Value in Health*, 22(9)).

Note. Converted to Euro by the rate of €1 = 11.48 Swedish krona. Price level 2023.

Abbreviations. RCC – Regional Cancer Center Stockholm-Gotland. SCB – Statistics Sweden. TLV – Swedish Dental and Pharmaceutical Benefits Agency. KPP – Swedish Cost per patient database.

Table A2. Distribution of costs

	Mean(€/ person)	Mini-mum	25th perc.	Median	75th perc.	Maxi-mum
Annual costs of screening						
Sum of costs for FOBT	8.46	0.94	4.51	10.74	10.74	120.92
Sum of costs for colonoscopy	18.91	0	0	0	0	2509.95
Sum of healthcare costs	27.38	1.72	4.51	10.74	10.74	2510.89
Sum of costs to wider society	4.02	0	0	0	0	497.08
Total costs, societal perspective	31.40	1.72	4.51	10.74	10.74	3007.97
Five-rounds-screening program						
Sum of costs for FOBT	37.09	3.73	22.37	40.60	49.63	126.80
Sum of costs for colonoscopy	58.98	0	0	0	0	3902.88
Sum of healthcare costs	96.07	3.73	22.37	42.85	50.15	3950.66
Sum of costs to wider society	13.72	0	0	0	0	873.71
Total costs, societal perspective	109.78	3.73	22.37	42.85	50.15	4810.11

Table A3. Annual costs of screening, grouped by participation-test results category

	Number per 10,000 people	Mean cost	St dev	Annual costs per 10,000 people
True positive (CRC or AA)	70			
Sum of costs for FOBT		8.93	3.27	625.58
Sum of costs for colonoscopy		1,437.38	562.79	100,702.07
Sum of other costs to society		223.20	76.18	15,637.46
Total costs, societal perspective		1,669.51	630.40	116,965.10
False positive	112			
Sum of costs for FOBT		8.89	3.89	996.74
Sum of costs for colonoscopy		788.59	322.36	88,410.31
Sum of other costs to society		219.36	81.67	24,593.10
Total costs, societal perspective		1,016.85	398.57	114,000.10
Negative test	6452			
Sum of costs for FOBT		10.64	2.42	68,644.96
Sum of costs for colonoscopy		0.00	0.00	0.00
Sum of other costs to society		0.00	0.00	0.00
Total costs, societal perspective		10.64	2.42	68,644.96
Non-participant	3270			
Sum of costs for FOBT		4.04	0.98	13,200.96
Sum of costs for colonoscopy		0.00	0.00	0.00
Sum of other costs to society		0.00	0.00	0.00
Total costs, societal perspective		4.04	0.98	13,200.96
Incomplete test	73			
Sum of costs for FOBT		12.96	8.48	945.25
Sum of costs for colonoscopy		0.00	0.00	0.00
Sum of other costs to society		0.00	0.00	0.00
Total costs, societal perspective		12.96	8.48	945.25
Positive, no colonoscopy	23			
Sum of costs for FOBT		9.98	2.43	232.23
Sum of costs for colonoscopy		0.00	0.00	0.00
Sum of other costs to society		0.00	0.00	0.00
Total costs, societal perspective		9.98	2.43	232.23

Table A4. Five-rounds-screening program: Selected resources per 10 000 screening occasions, by screening round and by test type

	Overall	By screening round					By test type	
		Round1	Round2	Round3	Round4	Round5	gFOBT	FIT
n	427,695	92,693	89,491	86,006	82,146	77,359	243,614	184,081
Test kits, gFOBT	5,696.0	9,986.6	9,810.0	5,114.1	2,345.8	..	10,000	..
Test kits, FIT	4,304.0	13.4	190.0	4,885.9	7,654.2	10,000	..	10,000
Re-test kits	462.0	521.6	623.9	463.6	355.5	314.5	588.2	294.9
Reminders	4,740.3	5,898.0	5,449.4	4,550.5	3,924.1	3,610.7	5,516.3	3,713.4
Lab analyses	6,271.3	5,351.5	5,605.6	6,387.5	6,915.4	7,330.6	5,605.5	7,152.5
No. of colonoscopies								
…without findings	61.9	64.9	53.4	68.3	61.0	62.0	62.4	61.3
…with biopsy	8.5	10.2	7.0	10.5	7.7	6.6	8.9	7.8
…with polypectomy	65.0	40.2	38.3	69.2	78.6	106.3	40.6	97.2
Findings to PAD	62.6	43.2	39.8	61.9	70.7	104.3	42.2	89.5

Note. gFOBT were used up until 2015, after which FIT were used.

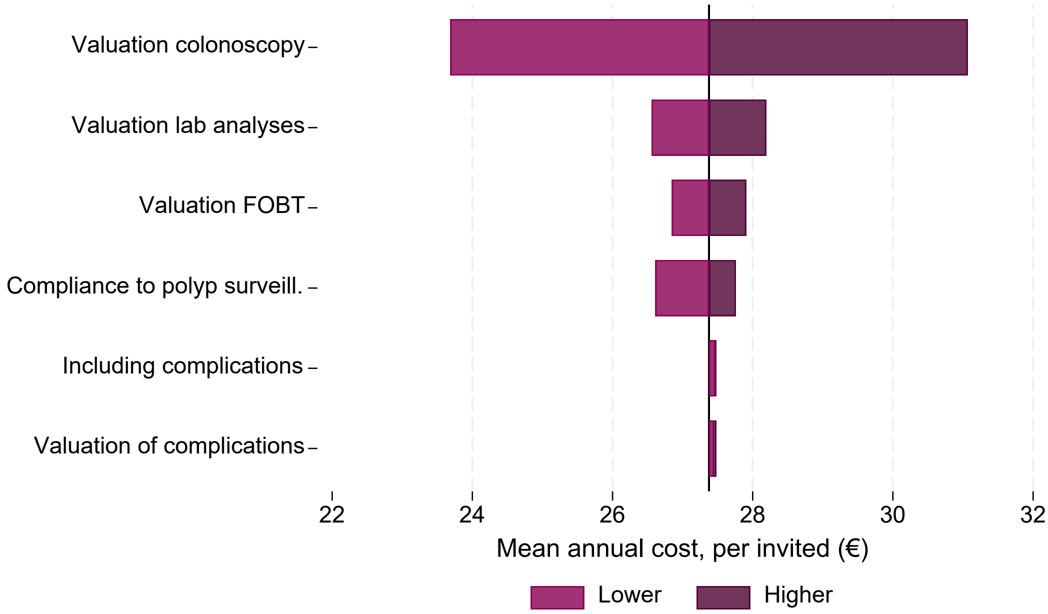

Figure A1. Deterministic sensitivity analysis – Annual costs of screening.

Note. Valuations and assumptions varied one at a time: Valuation of colonoscopy with and without biopsy or polypectomy varied ±20 percent from base case, valuation of lab analyses ±20 percent, valuation of FOBTs ±20 percent. Compliance to polyp surveillance program varied from 0.7 to 1.0 (0.9 in base case). Complications were included using indicator for any complication with value of €6650 (Arrospide et al 2018) and varied with +20 percent, and valuation of complications varied ±20 percent.

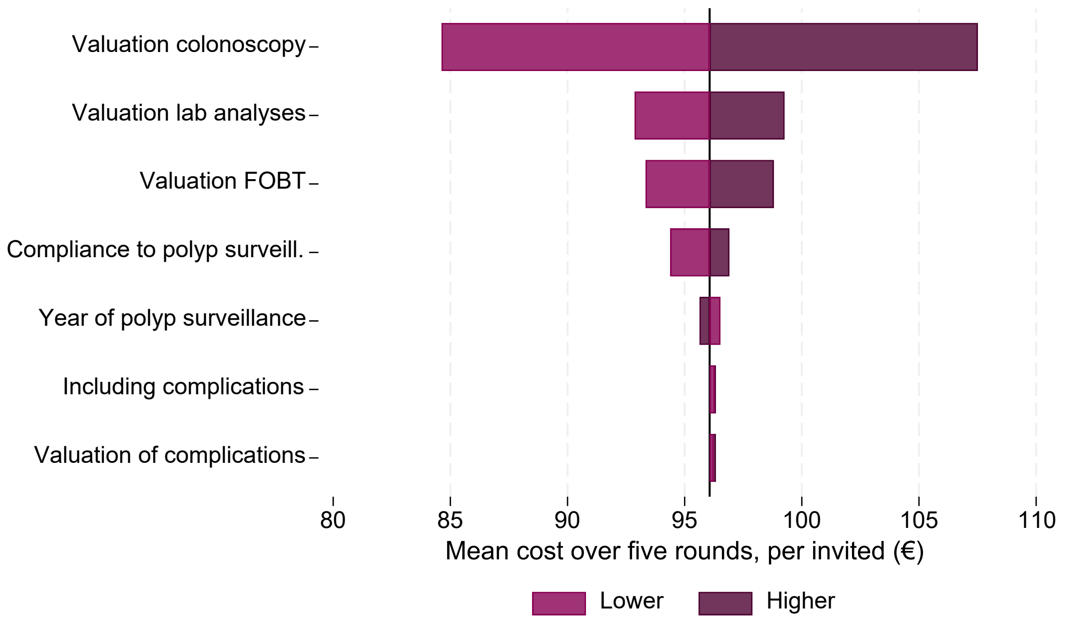

Figure A2. Deterministic sensitivity analysis – Costs for five-rounds-screening program.

Note. Valuations and assumptions varied one at a time: Valuation of colonoscopy with and without biopsy or polypectomy varied ±20 percent from base case, valuation of lab analyses ±20 percent, valuation of FOBTs ±20 percent. Compliance to polyp surveillance program varied from 0.7 to 1.0 (0.9 in base case), year of polyp surveillance colonoscopy varied from 1 to 5 years (3 years in base case). Complications were included using indicator for any complication with value of €6650 (Arrospide et al 2018) and varied with +20 percent, and valuation of complications varied ±20 percent. Costs were discounted.
